# Supplementary material for: Recombinant Thaumatin-Like Protein (rTLP) and Chitinase (rCHI) from Vitis vinifera as Models for Wine Haze Formation
Source: Molecules. 2022 Sep 28;27(19):6409. doi: 10.3390/molecules27196409 (PMC9573663; doi:10.3390/molecules27196409)
Supplement: Supplementary file 1 [file molecules-27-06409-s001.zip › molecules-1929718-supplementary.pdf]

Supplementary information

Title

Recombinant thaumatin-like protein (rTLP) and chitinase (rCHI) from *Vitis vinifera* as models for wine haze formation

Authors

Wendell Albuquerque [1], Pia Sturm [1], Quintus Schneider [1], Parviz Ghezellou [2], Leif Seidel [3], Daniel Bakonyi [4], Frank Will [3], Bernhard Spengler [2], Holger Zorn [1,4], Martin Gand [1*]

Affiliations

[1]Institute of Food Chemistry and Food Biotechnology, Justus Liebig University Giessen, Heinrich-Buff-Ring 17, 35392 Giessen, Germany

[2]Institute of Inorganic and Analytical Chemistry, Justus Liebig University Giessen, Heinrich-Buff-Ring 17, 35392 Giessen, Germany

[3]Department of Beverage Research, Geisenheim University, Von-Lade-Straße 1, 65366 Geisenheim, Germany

[4]Fraunhofer Institute for Molecular Biology and Applied Ecology, Ohlebergsweg 12, 35392 Giessen, Germany

*Corresponding author:

E-Mail: Martin.Gand@lcb.chemie.uni-giessen.de, ORC-ID: 0000-0001-8211-691X

Supplementary information includes:

Supplementary Figures S1 to S22

Supplementary Tables S1 to S13

Supplementary Materials and Methods

Supplementary Results and Discussion

Supplementary References

1. Supplementary Material and Methods

1.1. Cloning and transformation

1.1.1. Plasmid maps of the vectors pPIC9K harboring a TLP (UniProt ID: F6HUG9) and a class IV chitinase (UniProt ID: Q7XAU6)

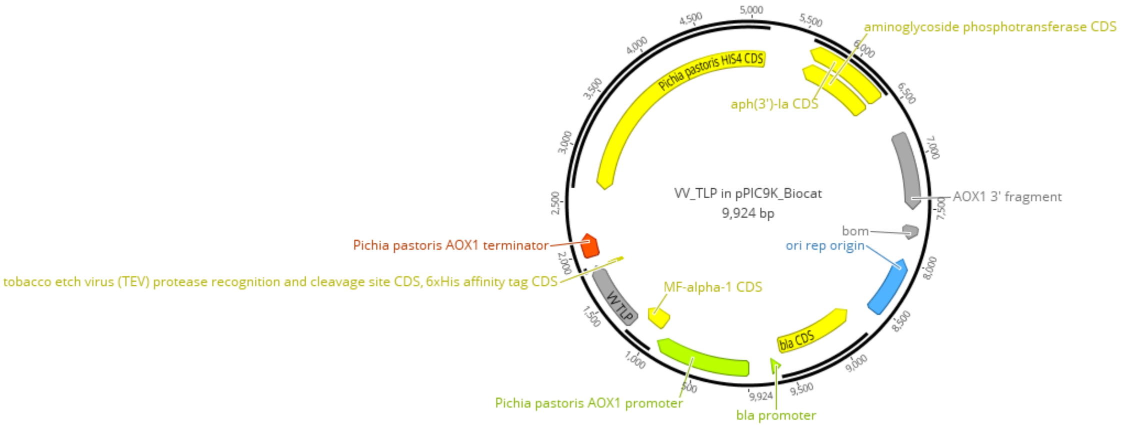

Figure S1: Plasmid vector pPIC9K encoding a thaumatin-like protein (Uniprot ID: F6HUG9).

Table S1: Genes and motifs in the plasmid pPIC9K-TLP (UniProt ID: F6HUG9). CDS = coding DNA sequence

Name	Type	Minimum	Maximum	Length	Direction
**bla promoter**	Promoter	9,615	9,719	105	reverse
**bla CDS**	CDS	8,754	9,614	861	reverse
**ori rep origin**	Origin	7,995	8,583	589	reverse
**bom**	Mobility region from pBR322	7,798	7,938	141	forward
**aminoglycoside phosphotransferase**	CDS	5,576	6,391	816	reverse
**6xHis affinity tag**	CDS	1,990	2,007	18	forward
**Tobacco etch virus (TEV) protease recognition and cleavage site**	CDS	1,963	1,983	21	forward
***Pichia pastoris* HIS4**	CDS	2,628	5,162	2,535	reverse
***Pichia pastoris* AOX1**	Terminator	1,969	2,215	247	forward
**VV TLP**	CDS	1,222	1,889	668	forward
**MF-alpha**	Secretion signal	949	1,215	267	forward

Table S2: Amino acid sequences of the integrated in the plasmids pPIC9K. a) TLP (UniProt ID: F6HUG9) and b) from a native TLP from *V. vinifera*

a) TLP (UniProt ID: F6HUG9) integrated in the plasmid vector pPIC9K
**EFMATFNIQNHCSYTVWAAAVPGGGMQLGSGQSWSLNVNAGTTGGRVWARTNCNFDASGNGKCETGDCGGLLQCTAY GTPPNTLAEFALNQFSNLDFFDISLVDGFNVPMAFNPTSNGCTRGISCTADIVGECPAALKTTGGCNNPCTVFKTDEYCCNSGS CSATDYSRFFKTRCPDAYSYPKDDQTSTFTCTAGTNYEVVFCPENLYFQSAGHHHHHH***
***N*-terminal part of the MF-α secretion signal, TEV protease recognition sequence and 6x-histidine tag sequence**
b) TLP (UniProt ID: F6HUG9) from *V. vinifera*
**MGLCKILSISSFLLTALFFTPSYAATFNIQNHCSYTVWAAAVPGGGMQLGSGQSWSLNVNAGTTGGRVWARTNCNFDASG NGKCETGDCGGLLQCTAYGTPPNTLAEFALNQFSNLDFFDISLVDGFNVPMAFNPTSNGCTRGISCTADIVGECPAALKTTGG CNNPCTVFKTDEYCCNSGSCSATDYSRFFKTRCPDAYSYPKDDQTSTFTCTAGTNYEVVFCP***
N-terminal peptide sequence

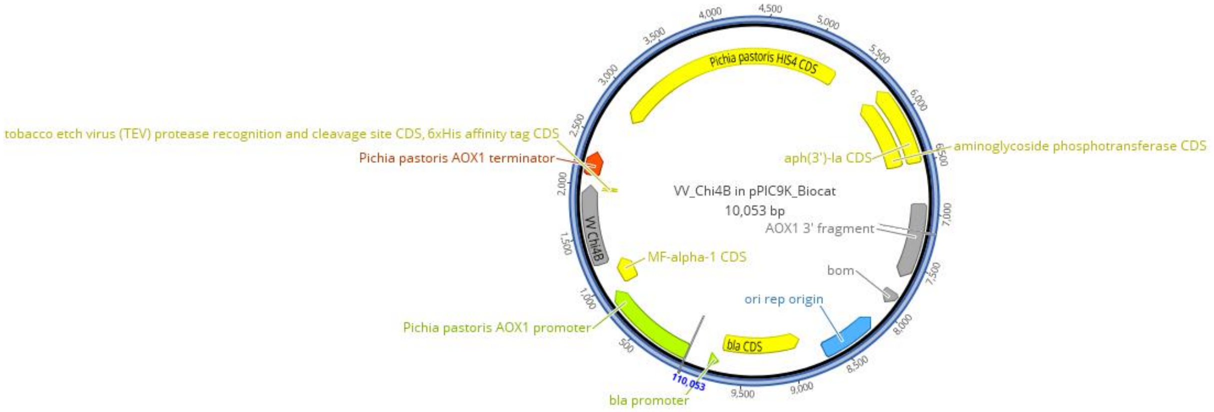

Figure S2: Plasmid vector pPIC9K encoding a class IV chitinase (UniProt ID: Q7XAU6).

Table S3: Genes and motifs in the plasmid pPIC9K-CHI (UniProt ID: Q7XAU6). CDS = coding DNA sequence

Name	Type	Minimum	Maximum	Length	Direction
**bla**	CDS	8,883	9,743	861	reverse
**bla promoter**	Promoter	9,744	9,848	105	reverse
**ori rep origin**	Origin	8,124	8,712	589	reverse
**bom**	Mobility region from pBR322	7,798	7,938	141	forward
**aminoglycoside phosphotransferase**	CDS	5,705	6,520	816	reverse
**6xHis affinity tag**	CDS	1,990	2,007	18	forward
**Tobacco etch virus (TEV) protease recognition and cleavage site**	CDS	1,963	1,983	21	forward
***Pichia pastoris* HIS4**	CDS	2,757	5,291	2,535	reverse
***Pichia pastoris* AOX1**	Terminator	2,098	2,344	247	forward

**VV Chi4B**	CDS	1,222	2,018	797	forward
**MF-alpha**	Secretion signal	949	1,215	267	forward

Table S4: Amino acid sequence of a CHI (UniProt ID: Q7XAU6). a) integrated in the plasmid pPIC9K and b) from a native CHI from *Vitis vinifera*

a) CHI (UniProt ID: Q7XAU6) integrated in the plasmid vector pPIC9K

**EFMQNCGCASGLCCSKYGYCGTGSDYCGDGCQSGPCDSSSGSGSSVSDIVTQSFFDGIINQAASSCAGKNFYTRAAFLSALNS
YSGFGNDGSTDANKREIAAFFAHVTHETGHFCYIEEINGASHNYCDSSNTQYPCVSGQNYYGRGPLQLTWNYNYGAAGNSIG
FNGLSNPGIVATDVVTSFKTALWFWMNNVHSVIGQGFGATIRAINGAVECNGGNTAAVNARVQYYKDYCSQLGVSPGDNL
TCENLYFQSAGHHHHHH***

***N*-terminal part of the MF-α secretion signal, TEV protease recognition sequence and 6x-histidine tag sequence**

b) CHI (UniProt ID: Q7XAU6) from *V. vinifera*

**MAAKLLTVLLVGALFGAAVAQNCGCASGLCCSKYGYCGTGSDYCGDGCQSGPCDSSSGSGSSVSDIVTQSFFDGIINQAASSC
AGKNFYTRAAFLSALNSYSGFGNDGSTDANKREIAAFFAHVTHETGHFCYIEEINGASHNYCDSSNTQYPCVSGQNYYGRGPL
QLTWNYNYGAAGNSIGFNGLSNPGIVATDVVTSFKTALWFWMNNVHSVIGQGFGATIRAINGAVECNGGNTAAVNARVQ
YYKDYCSQLGVSPGDNLTC**

N-terminal peptide sequence

1.1.2. Plasmid isolation from *Escherichia coli* and transformation in *Komagataella phaffii*

Synthetic plasmids encoding a thermolabile thaumatin-like protein (UniProt ID: F6HUG9) and a class IV chitinase (UniProt ID: Q7XAU6) were cloned into the vector pPIC9K between the *Eco*RI and *Not*I cleavage sites (performed by BioCat GmbH, Heidelberg, Germany) and were subsequently replicated in competent *E. coli* NEB 10-beta cells (New England Biolabs GmbH, Ipswich, MA, USA). For that, competent *E. coli* cells were picked from stocks and transferred to 5 mL of lysogeny broth (LB)-ampicillin medium [1% tryptone (w/v), 0.5% yeast extract (w/v), 1% NaCl (w/v) and 0.01% ampicillin (all purchased from Carl Roth, Karlsruhe, Germany)]. Cultures were grown overnight at 37 °C in shakers set at 180 rpm. To set up the main culture, 100 mL of LB-Amp medium was inoculated with 1 mL of the previous overnight culture and then incubated at 180 rpm and 37 °C for 6 h. Afterwards, cells were harvested by centrifugation (4,000 × g for 30 min). The cell pellets obtained were stored at -20 °C until further use. Plasmids present in the remaining 4 mL overnight culture were isolated using the FastGene Plasmid Mini Kit from Nippon Genetics (Nippon Genetics Europe GmbH, Düren Germany) and sequenced by Sanger sequencing (Eurofins Genomics Germany GmbH, Ebersberg, Germany).

Invitrogen's PureLink™ HiPure midiprep kit (Thermo Fisher Scientific Inc., Waltham, MA, USA) was used to extract plasmids from the stored cultures at -20 °C. Isolation was performed according to the midiprep batch procedure outlined in the user manual. The samples were subsequently dried under a stream of nitrogen, added to 110 µL TE buffer (from the kit) and stored at -20 °C. To check the DNA concentration, the plasmid extracts were measured on the nanoPhotometer™ (Implen, Munich, Germany).

1.1.3. Restriction digestion of the extracted plasmid DNA

In order to linearize the plasmid DNA, a restriction digestion was carried out with the enzymes *Bgl*II and *Sac*I (about 52 µL of the plasmid material, 6 µL of digestive enzyme solution and 2 µL of digestion buffer) at temperatures of 37 °C for 2 h, 65 °C for 10 min and subsequently cooled at 15 °C. The digested plasmids were analyzed by means of agarose gel electrophoresis [1% Tris-acetate-EDTA (TAE) agarose gel]. After electrophoresis, the desired gel fragments were cut out of the gel using a scalpel, transferred to empty 1.5 mL reaction tubes and weighed.

1.1.4. Extraction of plasmid DNA from gel fragments and electroporation

The DNA in the gel fragments was extracted using the Macherey-Nagel NucleoSpin® Gel and PCR Clean up Kit (Düren, Germany). The procedure was mainly based on the manufacturer's user manual. The DNA concentration was determined using a nanophotometer and agarose gel electrophoresis was carried out to confirm proper extractions.

Colonies of *K. phaffii* strain GS115 (wild type) were picked from a yeast peptone dextrose (YPD) medium [yeast extract (10 g/L); peptone (20 g/L); glucose (20 g/L)] and agar (15 g/L agar), all purchased from Carl Roth)] and placed in a 250 mL baffled flask filled with 100 mL of YPD liquid medium. The flasks where then incubated at 30 °C and 200 rpm overnight. A volume of 1 mL of the overnight culture was transferred to a new 250 mL baffled flask filled with 100 mL YPD medium. This culture was also incubated at 30 °C and 200 rpm. During the incubation, 1 mL of the main culture was taken regularly to determine the optical density at 600 nm. After 1 h of incubation, another 5 mL of the overnight culture were added to the main culture and the optical density was measured immediately afterwards. Cells were then harvested by centrifugation (4,000 × *g*, 4 °C, 20 min) and the residual pellet was resuspended in 1 mL of ice-cold and sterile 1 M sorbitol and used directly for electroporation.

Electroporation cuvettes were filled with 80 µL of the yeast culture dissolved in sorbitol and added by 10 µL of linearized plasmid DNA solution. The cuvettes were then incubated on ice for 5 min and then transformed using an electroporator. The electroporation times were between 5.3 and 5.8 ms for all approaches. After electroporation, the samples were incubated for 1 h at 30 °C and finally treated with 1 mL of ice-cold, sterile sorbitol. 20 transformants for each electroporation batch (TLP *Bgl*II; TLP *Sac*I; CHI *Bgl*II: CHI *Sac*I) were set, achieving a total of 80 clones tested.

1.1.5. Cultivation of transformed *K. phaffii* on MD without histidine, genetics and YPD agar plates

The success of the transformation/electroporation was evaluated by growing the clones in histidine-deficient agar plates. For this purpose, 700 and 300 µL of the transformed cells were spread in agar plates with dextrose minimal medium without histidine (MD-His) using a *Drigalski* spatula. The agar plates were then incubated at 30 °C for about 72 h. After the incubation period, the cell cultures on the plate were counted.

For testing the antibiotic resistance associated to the integrated gen in the vector pPIC9K, geneticin was concentrated at 0.5, 1, 1.5 and 2 mg/mL in agar plates. Electroporation batches from MD-His agar plates were mixed with 1.3 mL of sterile water and pipetted (250 µL) on the geneticin plate and spread using a *Drigalski* spatula. The plates were then incubated at 30 °C for 96 h.

For stable storage of the clones after screening for antibiotic resistance, they were transferred to YPD agar plates. For this purpose, the were picked from geneticin agar plated and inoculated YPD agar plates.

1.1.6. Phenotype determination: Cultures on MM and MD agar plates and PCR amplification of *AOX1* gen

To determine the phenotype, two types of agar plates were prepared from different minimal media with dextrose (MD) [1.34% Yeast Nitrogen Base YNB (VWR International GmbH, Darmstadt, DE), 0.00004% biotin (Carl Roth) and 1% glucose (Carl Roth)] or methanol (MM) (1.34% YNB, 0.00004% biotin and 0.5% methanol (J.T. Baker, Gliwice, Poland). Subsequently, individual clones were picked from YPD agar plates and transferred onto MD and MM agar plates, which were incubated at 30 °C for 48 h.

For the amplification of *AOX1* gen, individual clones were picked from a YPD agar plate and transferred to a well of a PCR plate previously filled with 20 µL of 0.2 M sodium hydroxide (Carl Roth) solution. The PCR plate was then heated in a thermal cycler at 99 °C for ten min to support cell lysis. The lysate was then centrifuged (4,000 × g, 4 °C, 10 min). For the PCR analysis, 1 µL of the supernatant obtained was pipetted into 20 µL of a master mix solution (4 µL of 5x OneTaq Standard Buffer, 0.08 µL of 100 mM dNTPs, 1.60 µL of Pichia_AOX1 3´-Primer (AOX1_Fw 3'-GACTGGTTCCAATTGACAAGC-5'), 1.60 µL of Pichia_AOX1 5´-Primer (AOX1_Rv 5'-GCAAATGGCATTCTGACATCC-3'), 13.76 µL of sterile water and 0.16 µL of OneTaq-Polymerase) (primers were purchased from Biomers.net, Ulm, Germany) and added into a new PCR plate and followed PCR standard protocols.

A volume of 5 µL of the sample or marker was mixed with 1 µL of Midori green DNA stain (Nippon Genetics Europe GmbH) and run in 1% TAE agarose gel for 45 to 60 min at 100 V. The 1 kbp standard marker (Nippon Genetics Europe GmbH) was used as a DNA marker. Agarose gels were digitally photographed under ultraviolet (UV) light.

1.2. Protein expression by *K. phaffii*

1.2.1. Fermentations

Cells were picked from YPD agar plates to inoculate 50 mL of buffered complex glycerol (BMGY) medium (1% yeast extract; 2% peptone; 100 mM potassium phosphate, pH 6.0; 1.34% YNB; 0.00004% biotin; 1% glycerol or 0.5-1% methanol) in Erlenmeyer flasks (250 mL) during overnight incubation at 200 rpm and 30 °C. For the main cultures, 500 mL baffled flasks, filled with 100 mL buffered complex methanol (BMMY) medium (1% yeast extract; 2% peptone; 100 mM potassium phosphate, pH 6.0; 1.34% YNB; 0.00004% biotin; 1% methanol), were inoculated with 1 mL of the previous overnight culture and incubated at 200 rpm and 30 °C. To enable constant expression, methanol (1%) was supplied every 24 h. The optical density of the main cultures was also determined every 24 h. Culture supernatants were concentrated using 10 kDa molecular weight cut-off filters (MWCOF, Merck KGaA, Darmstadt, Germany) by a factor of about 200 and were stored at -20 °C.

1.2.2. Upscaling expression of rTLP and rCHI

To upscale the protein expression, transformed *K. phaffii* transformed cells were cultured in 600 mL of BMMY medium were filled into a 2 L baffled flask and inoculated with 4 mL of overnight cultures. Methanol was fed (1% of the fermentation volume) daily. The resulting supernatant was concentrated using pressure-driven dialysis over a 10 kDa MWCOF integrated polyethersulfone (PES) membrane. Centrifuge tubes with an integrated 10 kDa MWCOF were used for further concentration. The samples were concentrated by a factor of 200 to 400, and stored at -20 °C.

1.2.3. Protein content

The protein content was quantified according to Bradford et al. [1] and following standard protocols of a ROTI® Nanoquant reagent (Carl Roth) using bovine standard albumin (BSA, Carl Roth) as standard.

1.2.4. SDS-PAGE

SDS-PAGE was performed in denaturing conditions according to Laemmli et al. [2] using a 12% resolving gel. Samples were previously heated at 80 °C for 10 min and mixed with a denaturing buffer containing β-mercaptoethanol (Carl Roth) as denaturing agent and bromophenol blue as dye. A PageRuler™ Prestained (Thermo Fischer Scientific Inc) was used as marker. Electrophoresis was performed at 20 mA and gels were stained by a Coomassie blue staining (Coomassie Brilliant Blue-R250, AppliChem, Darmstadt, Germany) solution and gels were photographed using a Microtek's Bio-5000 scanner (Kapelan Bio-Imaging, Leipzig, Germany).

1.2.5. Western blot (WB)

Proteins separated by SDS-PAGE (including protein markers) were blotted in polyvinylidene fluoride (PVDF) membranes (Carl Roth) following standard Western Blot (WB) procedures. Blotting was performed at 100 V for 1 h, per gel. After blotting, the membranes were washed once in 15 to 20 mL of TBS-T buffer, incubated for 1 h at 4 °C in a 5% milk powder TBS-T solution. The PVDF membrane was then incubated for 2 h in a primary 6x-His tag monoclonal antibody (HIS.H8) (Thermo Fischer Scientific Inc) at 4 °C and subsequently incubated with a secondary anti-Mouse IgG antibody (Thermo Fisher Scientific Inc) for 2 h at 4 °C. The Opti-Dilut 4CN (Bio-Rad Laboratories, Munich, Germany), substrate kit was used for detection of his-tagged proteins.

1.3. Purification of the expressed proteins

1.3.1. Immobilized Metal Affinity Chromatography (IMAC)

His-tagged proteins were purified by IMAC chromatography. Isocratic flows of the equilibration buffer A [50 mM sodium phosphate, 300 mM NaCl (purchased from Carl Roth), pH 7] were followed by a 100% isocratic flow of buffer B used for elution (50 mM sodium phosphate, 300 mM NaCl, 250 mM imidazole (Carl Roth). The eluted protein fractions were then concentrated and simultaneously desalted using centrifuge tubes with an integrated 10 kDa MWCOF.

1.3.2. Size Exclusion Chromatography (SEC)

For further purification of rCHI and rTLP imidazole-eluted peaks collected from the IMAC were subjected to a HiLoad 16/600 Superdex 75 column (Cytiva Europe GmbH, Freiburg, Germany), aiming to separate proteins per molecular weight (MW) at 1 mL/min using 0.1 M Tris-HCl buffer (pH 7) as eluent.

Fractions were pooled and concentrated by 10 kDa MWCOF and then analyzed qualitatively by SDS-PAGE with Coomassie staining and WB. A standard mix of proteins (Gel filtration protein standards, Bio-Rad Laboratories) consisting of thyroglobulin, γ-globulin, ovalbumin, myoglobin and vitamin B_{12} was used to compare the elution per molecular weight (MW) to the chromatogram retention time (RT).

1.4. Mass spectrometric analysis of the expressed rTLP and rCHI

1.4.1. MS-based bottom-up proteomics (sample preparation)

Purified proteins separated by SDS-PAGE and were excised from gels with a sterile scalpel and the gel fragments were then cut into small cubes and transferred to reaction tubes cleaned with 50% acetonitrile (ACN) (Merck KGaA)/1% trifluoroacetic acid (TFA) (Thermo Scientific Scientific Inc). For decolorization, the gel fragments were incubated twice for 45 min at 37 °C in 200 µL of 100 mM ammonium carbonate $(NH_4)_2CO_3$ (Thermo Scientific Scientific Inc)/50% ACN. The supernatant was disposed, and the gel fragments were dehydrated with 100 µL ACN for 5 min at room temperature and dried in a vacuum centrifuge.

Afterwards, proteins in the gel fragments were reduced (with 200 µL of 10 mM dithiothreitol (DTT) (Merck KGaA)/25 mM $(NH_4)_2CO_3$ for 30 min at 65 °C) and alkylated (with 200 µL of 10 mM iodacetamide (IAA) (Serva Electrophoresis, Heidelberg, Germany)/25 mM NH_4HCO_3 for

45 min at 37 °C). For trypsin digestion (according to the user manual from Promega GmbH, Walldorf, Germany), the gel fragments were mixed with 30 µL trypsin/Lys C solution at 37 °C for 16 h. To extract the peptides, the gel fragments were incubated with 50 µL of 50% ACN/5%TFA (in water) for 60 min at room temperature and afterwards the residual liquid phase was transferred to another reaction tube and dried. Samples were further desalted and concentrated using standards ZipTips (Merck KGaA) protocols.

1.4.2. MS-based bottom-up proteomics (LC-MS Analysis)

The peptides were separated using an UltiMate 3000 RSLC HPLC system (Ultra-High-Performance Liquid Chromatography, Thermo Fisher Scientific Inc) on a Kinetex C18 (2.1 × 100 mm, 2.6 µm 100 Å particle size) column (Phenomenex, Torrance, CA, USA) coupled to a Q Exactive HF-X (Thermo Fisher Scientific Inc) mass spectrometer. Chromatographic analysis was performed at 250 µL/min flow rate with water/0.1% formic acid (mobile phase A) and ACN/0.1% formic acid (mobile phase B). The optimized gradient elution of 90 min was applied as follows: isocratically (2% B) for 5 min, followed by 2-40% B over 70 min, 40-50% B over 5 min, 50-98% B over 2 min, and re-equilibration in 2% B. The mass spectrometers were operated in data-dependent acquisition (top-10 DDA) with the following parameters in full MS scans: mass range of m/z 350 to 1800, resolution of 120,000 (at m/z 200), AGC target of 3e6, IT of 50 ms, and MS/MS scans: mass range of m/z 200 to 2000, mass resolution of 30,000 (at m/z 200), AGC target of 1e5, IT of 120 ms, isolation window m/z 1.3 and dynamic exclusion of 60s. The data were analyzed using the software of Proteome Discoverer version 2.5 (Thermo Fisher Scientific Inc) against protein sequences (proteome of *V. vinifera*) downloaded from UniProt and NCBI databases. Target-decoy statistical analysis was performed with false discovery rate (FDR) of 1%.

1.4.3. MS-based proteomics (glycosylation analysis)

Tryptic digested protein fragments from rTLP and rCHI were further analyzed by the software SimGlycan v. 5 (PREMIER Biosoft, Palo Alto, CA, USA) (Meitei et al. 2015). For that, the MS/MS data related to the purified rTLP and rCHI was uploaded to the software and the peak search was performed through the tools "peak detection and picking" with RT tolerance of 0.1%; m/z tolerance of 0.01 Da; charge state 1 to 3 and isotopic peak M+2 to M+6. The glycan search was performed by using the tool "glycopeptide qualitative analysis" with monosaccharides from

the Simglycan database. The protein sequences were the same as shown in the Table S3 and S4, for rTLP and rCHI, respectively.

1.5. Chitinolytic activity

The chitinolytic potential of the rCHI was tested by degrading chitin [prepared as described by Trudel and Asselin et al. [3] embedded in agarose gel according to Zou et al. [4], with modifications. For that, chitin from crab shells (Carl Roth) was firstly hydrolyzed by 1 M HCl (for 4 h at 4 °C), added by ethanol and centrifuged (4,000 × *g*, 10 min, 4 °C*)*. The precipitated "gel" formed was frozen at -80 °C, lyophilized and resuspended in 0.1 M TRIS-HCl buffer (pH 7). The chitin solution (roughly estimated at 0.1 g/mL) [added by 10 µL ampicillin (stock solution of 10 mg/mL)], was mixed (50%, v/v) with a boiling agarose (2%) solution (Biozym Scientific GmbH, Hessisch Oldendorf, Germany, dissolved in 0.1 M TRIS-HCl buffer pH 7) and poured in Petri dishes. After the gel polymerization, circular holes (about 0.5-1 cm diameter) were made in the center of the gels and aliquots of 20 µL of different samples were added: a) the purified rCHI (0.5 mg/mL); b) a native (non-heated) CHI (0.5 mg/mL) from *Streptomyces griseus* (Merck KGaA, used as positive control); c) a denatured (heated at 80 °C) CHI (0.5 mg/mL) from *S. griseus* (0.5 mg/mL) (used as negative control); and d) the lyophilized protein content of a Silvaner Franken wine (in 0.1 M Tris-HCl buffer pH 7) (used as comparative control); followed by overnight incubation at 37 °C. Halos of degradation were revealed after incubation (10 min) with calcofluor white stain (0.1 g/mL, Merck KGaA) and photographed after a washing step with distilled water (in orbital shaker with 25 rpm for 1 h) and left to rest for 1 hour at room temperature. In addition, a quantitative estimation of the chitinolytic activity was performed as described by Breuil and Saddler [5] and Brandt et al. [6] by DNS (3,5-dinitrosalicylic acid) assays. Briefly, 50 µL of the same enzymatic reaction's samples used in the agar diffusion experiments (described above) were mixed with 150 µL of solutions of hydrolyzed [with 37% HCl (Carl Roth)] chitin (1 mg/mL) and chitosan (AZCL-chitosan, Megazyme, Sidney, Australia, 1 mg/mL) and incubated at 37 °C for 3 h. Afterwards, 200 µL of 11 mM DNS reagent was added and the samples were incubated at 100 ˚C in water bath for 10 min. Aliquots of 150 µL were pipetted into 96 well plates and had optical densities (OD) measured at 575 nm. All assays were performed in triplicate and a standard curve was performed by using different concentrations of *N*-acetylglucosamine (Merck KGaA).

1.6. Extraction and analysis of polyphenols from grape (*V. vinifera*) juices

1.6.1. Polyphenol extracts

A chromatography glass column (250 mL) filled with XAD16 adsorber (Merck KGaA) resin was initially washed with 5 L water. A volume of 2.5 L of white grape juice (Niehoffs-Vaihinger Fruchtsaft GmbH, Lauterecken, Germany) was added to the column and subsequently washed with 5 L of water. Elution was done with 250 mL methanol, which was later removed on a Hei-Vap Value Digital rotary evaporator (Heidolph Instrumemts GmbH & Co. KG, Schwabach, Germany). After removing the ethanol, the solution was dried by lyophilization. Polyphenol extracts was dissolved in water with 1% methanol and concentrated at 2 mg/mL.

1.6.2. Analysis of polyphenols

The polyphenolic extracts were dried by lyophilization, dissolved in water (with 1% methanol) to a final concentration of 2 mg/mL. Samples (3 µL) were analyzed in a Thermo Scientific Ultimate 3000 UHPLC system and separated on a Luna 3u C18(2) column (Phenomenex, USA) using gradient flows (0.25 mL/min at 40 °C) of the eluent A [water (98%)/acetic acid (2%) and B [acetonitrile (49,75%)/water (49,75%)/acetic acid (0.5%)]. The concentrations of the eluents A and B were set as follows: at 0 min, A (96%)/B (4%); at 20 min, A (50%)/B (50%); at 23 min, A (0%)/B (100%); at 25 min, A (96%)/B (4%); at 31 min, A (96%)/B (4%)].

Chromatograms were obtained at 280, 320 and 360 nm and data processing was performed by the software Chromeleon^{TM} (Thermo Scientific Inc). External calibration was carried out by using the following polyphenols standards: Protocatechuic acid (Merck KGaA); Procyanidin B1, Procyanidin B2, Procyanidin C1 and quercetin-3-*O*-glucoside (Extrasynthese, Genay, France); Catechin, Epicatechin and coumaric acid (Fluka, Buchs, Switzerland); Caffeic acid and ferulic acid (Carl Roth).

1.6.3. Analysis of monosaccharides

Monosaccharides were determined after hydrolysis of the extract (10 mg) with 125 µL sulfuric acid (72%, Carl Roth) and 1,350 µL water at 120 °C in a test tube with cap [digestion block (Typ 2000) with temperature controller (TR-L 288, Liebisch, Bielefeld, Germany)] for 1 h. The samples were diluted (1:50) in water and filtered [filter with 0.2 µm pore size (VWR)]. Monosaccharides

were analyzed via HPAEC-PAD with a Carbo Pac PA-100 column (250 mm × 4 mm, Thermo Fisher Scientific Inc) coupled to a Dionex Bio-LC system. Neutral and acid sugars were analyzed separately. For both measurements 10 µL of the samples were injected and separated at a flow rate of 0.75 mL/min and a column temperature of 30 °C. Neutral and acid sugars were separated isocratically by 12 mM and 0.5 M sodium hydroxide solutions (Merck KGaA), respectively.

For quantification of monosaccharides, an external calibration was performed by using Fucose (AppliChem); Rhamnose, Arabinose, Xylose and Glucuronic acid (Merck KGaA); Galactose (Honeywell Inc., Charlotte, NC, USA); Glucose and Mannose (Carl Roth); Galacturonic acid (Alfa Aesar, Karlsruhe, Germany).

1.7. Comparison of the haze potential of the Silvaner Franken wine and rTLP and rCHI

The protein content from a lyophilized wine (described in https://doi.org/10.1016/j.foodchem.2021.130437) was solubilized in citrate buffer (pH 4) at a concentration of 0.2 mg/mL and subjected to a heat test. The influence of the polyphenols and sulfite ions were studied as described in the section 2.8.3 of the main manuscript. The heat test was performed according to Pocock and Waters [7] and the haze was measured at 540 nm, before and after sample centrifugation (12,000 × g for 10 min). Pellets formed after centrifugation were photographed and used for comparative analysis.

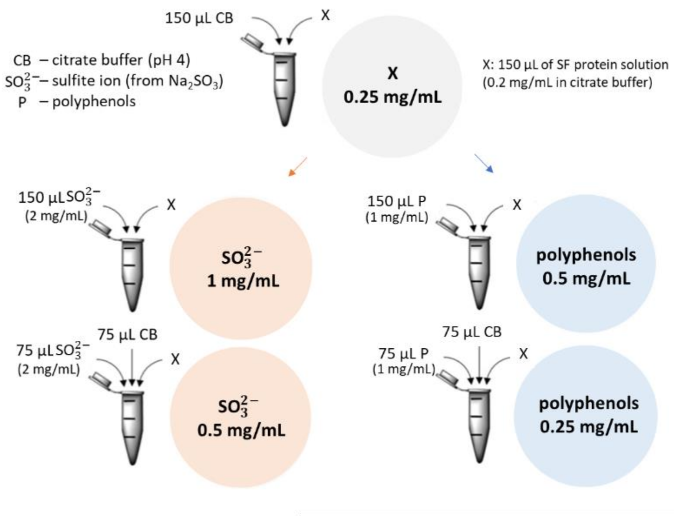

Figure S3: Overview of sample preparation for the heat tests, SF [solution of proteins (X) from a Silvaner Franken wine or rTLP or rCHI or combination of rCHI and rTLP] in combination with different concentrations of sulfite ions (SO_3^{2-}) and polyphenol extract (P) to identify their influence on the haze potential.

1.8. Bentonite fining

Bentonite fining was performed using bentonite clay (Merck KGaA) to test the adsorptivity potential of the rTLP and rCHI (and also a Silvaner Franken wine, for comparison), according to the methods described by Pocock et al. [8] and Pocock and Waters [7]. Bentonite powder was solved (5%, w/v) in boiling distilled water and subsequently diluted to three different concentrations (0.25, 0.5 and 1 g/L). A volume of 0.8 mL from each of the concentrations were mixed with 0.2 mL of the rTLP or rCHI solutions (0.5 mg/mL in 0.1 M citrate buffer pH 4). The samples were subsequently left to rest at room temperature for 2 h, centrifuged (1,500 × g for 20 min) and the supernatants were submitted to a heat test to confirm the removal of haze proteins.

2. Results

2.1. Plasmid isolation and transformation

Plasmids were successfully isolated from *E. coli* NEB 10-beta cells. The DNA concentrations (measured by nanophotometry) for the plasmids harboring the rTLP and rCHI were 657 ng/µL and 322 ng/µL, respectively. DNA bands in agarose gel were observed at around 10,000 bp for both plasmid vectors.

After isolation, the plasmids were linearized by the restriction enzymes *Bgl*II and *Sac*I, and the digested fragments were observed at about 3,000 and 8,000 bp were visible for the plasmid DNA

linearized with *Bgl*II (Figure S4). For the samples digested with *Sac*I only one band could be recognized on the agarose gel with a size about 10,000 bp (Figure S4).

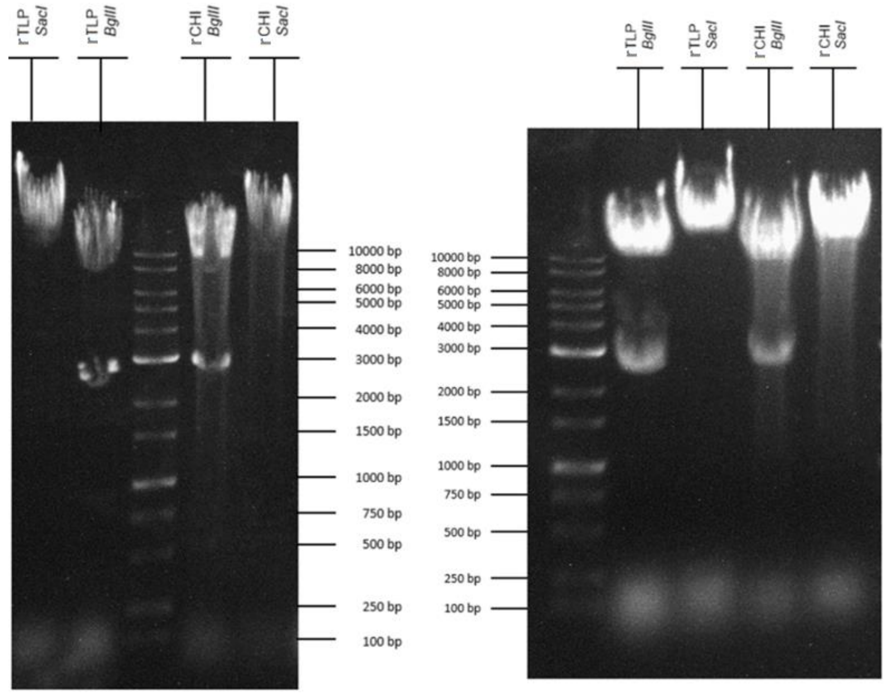

Figure S4: DNA separation of the digested plasmids DNA with *Bgl*II and *Sac*I in agarose gel.

After excision of the linearized plasmid DNA from the gel fragments, their concentrations were estimated as: rTLP (*Bgl*II)= 298 ng/µL; rTLP (*Sac*I)= 230 ng/µL; rCHI *Bgl*II= (246 ng/µL); rCHI *Sac*I= (208 ng/µL).

After electroporation, the transformants were plated on MD-His agar and cultivated as already described in the main manuscript (Section 2.2). The number of cell colonies is shown in the Table S5. The growth on MD-His agar could be observed in all electroporation approaches (Figure S5) and confirmed the successful integration of the expression cassette containing the *His4* gene into the *K. phaffii* genome.

Table S5: Colony counting after culturing on MD-His agar.

Sample	Volume plated [µL]	Colonies (n)
K. phaffii pPIC9k-rTLP *BglII*	300	292
	700	437

K. phaffii pPIC9k-rTLP *SacI*	300	762
	700	676
K. phaffii pPIC9k-rCHI *BglII*	300	111
	700	53
K. phaffii pPIC9k-rCHI *SacI*	300	42
	700	31

Growth on geneticin agar plates was observed in all electroporation approaches and high colony counts could be observed at different antibiotic concentrations (0.5, 1, 1.5 and 2 mg/mL as described in section 1.1.5). Figure S6 shows geneticin agar plates with transformants harboring the rTLP gene and linearized by the *BglII* restriction enzyme.

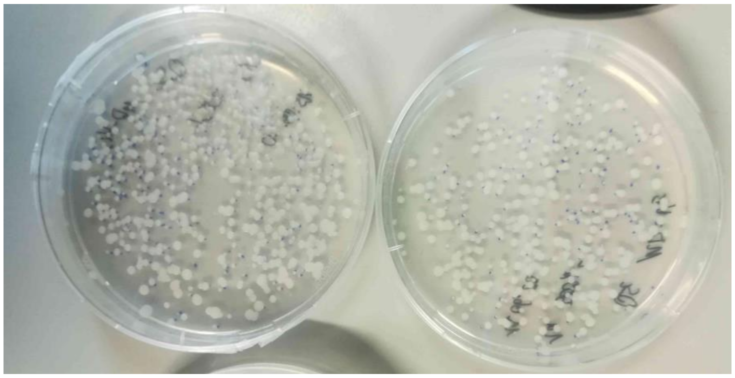

Figure S5: Transformed *K. phaffii* cells cultured on MD-his agar of the TLP *Bgl*II batch.

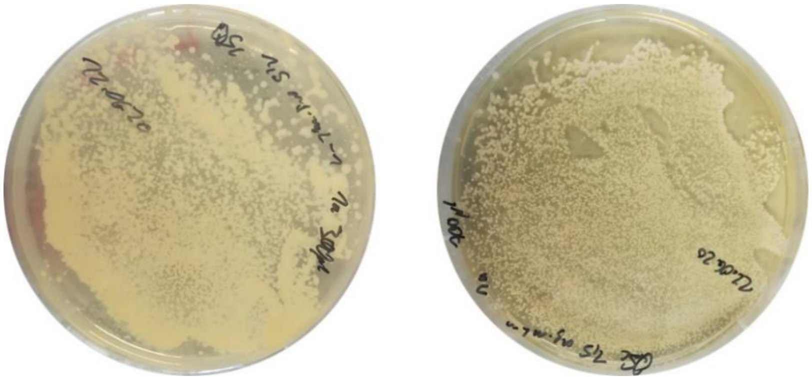

Figure S6: Transformed *K. phaffii* cells cultured on geneticin agar of the TLP *BglI*I batch.

2.2. Determination of the phenotype

2.2.1. Phenotype determination on MM and MD agar plates

In most clones, no significant differences were observed between the cell growth in agar plates with dextrose (MD) and methanol-minimal medium (MM) (Figure S7). Consequently, almost all

clones could only be assigned to the Mut+ phenotype, when the phenotype was determined using agar plates. Figure S7 shows transformants plated on MD and MM agar media.

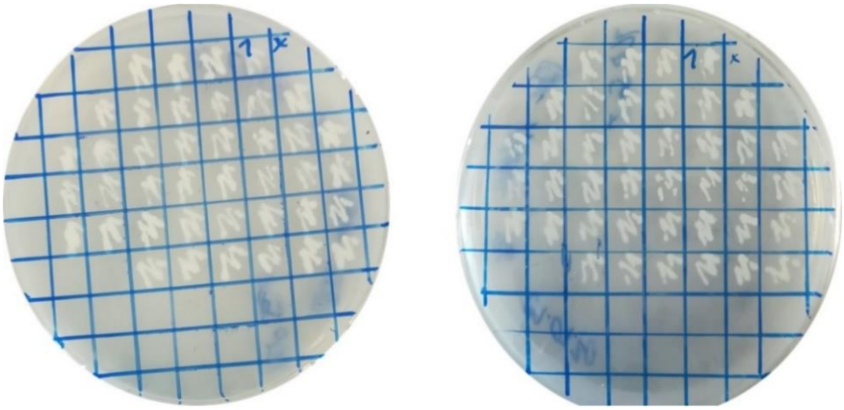

Figure S7: Cultivation on MD (left) and MM agar plates (right) of the Transformants of *K. phaffii* with the TLP *Sac*I batches.

2.2.2. *AOX1* gene determination using PCR

To evaluate a possible knock-out of the *AOX1* gene in the plasmids (methanol utilization slow-Mut^S phenotype), their amplicons were analyzed by electrophoresis (agarose gels). In the case of rTLP and rCHI the sizes of the PCR products were approximately 1,250 bp for the TLP amplicons or 1,500 bp for the CHI amplicons. In total, the presence of *AOX1* gene could be determined for 72 of 80 clones. The agarose gels containing the PCR products of the *AOX*1 amplification, are shown in the Figure S8. The integration of the *AOX1* gene in the plasmid pPIC9k confirms the Mut+ phenotype of the transformants.

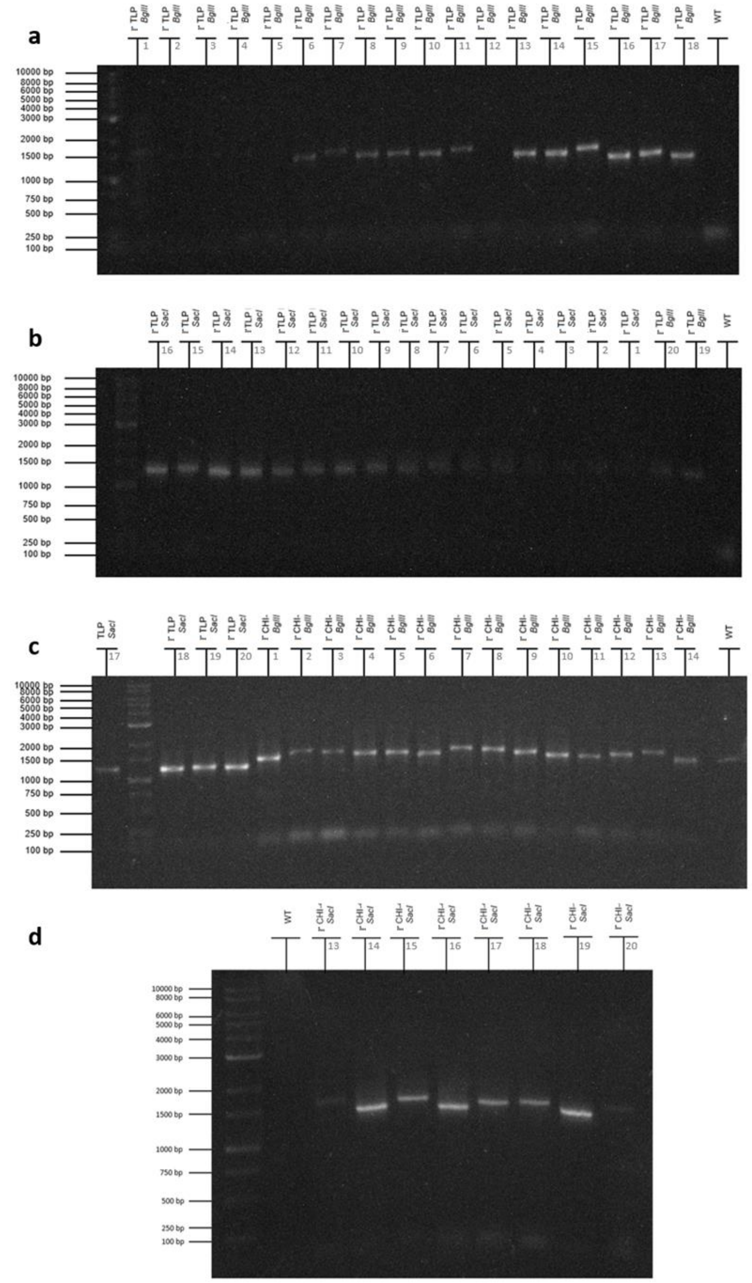

Figure S8: PCR products of the *AOX1* gene. a) rTLP (*Bgl*II batch); b) rTLP (*Sac*I batch); c) Mix of different electroporation batches TLP *Sac*I and CHI *Bgl*II); d) rCHI (*Sac*I batch).

2.3. Expression of 6x-his-tagged rTLP and rCHI

2.3.1 SDS-PAGE

Differences in the protein expression levels could be observed for rTLP and rCHI among all the clones. In clones harboring the rTLP, a dense band with an approximate size of 23 kDa could only be observed in two out of ten clones (Table S6) (clones 16 and 20 of the *Sac*I electroporation

batches). The bands were visible after 3-4 days of methanol induction. For rCHI, bands (double band) with a size of about 27 kDa-32 kDa could be observed in eight out of the ten clones used (Table S6), after 4 days of methanol induction. The list of the clones, which were able to express rTLP and rCHI are shown in the Table S6. Exemplary SDS-PAGE gels are shown in the Figure S9a.

Table S6: Protein expression visualized by SDS-PAGE after two and four days of induction.

protein	electroporation batch	clone (n)	**induction$_{2d}$**	**induction$_{4d}$**
rTLP	*BglII*	14	-	-
		15	-	-
		16	-	-
		17	-	-
		18	-	-
	SacI	14	-	-
		15	-	-
		16	X	X
		19	-	-
		20	X	X
rCHI	*BglII*	1	-	X
		4	X	X
		7	X	X
		14	X	X
		20	-	-
	SacI	4	-	-
		14	X	X
		15	X	X
		16	X	X
		17	X	X

Induction$_{2d}$: expression after two days of induction; Induction$_{4d}$: expression after four days of induction; X: expression observed; -: no expression observed

2.3.2. Western-Blot (WB)

Both His-tagged rTLP and rCHI could be detected by WB. In the case of rTLP, a band with a molecular weight of about 23 kDa could be observed for nine of the ten clones after two days of induction. Expression was also detected in four of these clones after four days of induction. Also for rCHI, bands with a molecular weight of approximately 27-35 kDa were visible in six of the ten clones (Table S7 and Figure S9). The most successful clones able to express rTLP were the clones 14, 15, 16 and 18 for the batch *Bgl*II, and the clones 14, 15, 16, 19 and 20 for the batch *Sac*I. rCHI

was expressed by the clones 1, 4, 14 and 20 for the batch *Bgl*II and by the clone 16 and 17 in the batch *Sac*I. The list of the expressed and detectable his-tagged proteins on WB membranes is showed in the Table S7.

Table S7: Protein expression examined by Western blot after two and four days of induction.

protein	electroporation batch	clone (n)	**induction$_{2d}$**	**induction$_{4d}$**
rTLP	*BglII*	14	X	-
		15	X	-
		16	X	X
		17	-	-
		18	X	X
	SacI	14	X	-
		15	X	-
		16	X	-
		19	X	X
		20	X	X
rCHI	*BglII*	1	X	-
		4	X	X
		7	-	-
		14	X	X
		20	X	X
	SacI	4	-	-
		14	-	-
		15	-	-
		16	X	X
		17	X	X

Induction$_{2d}$: expression after two days of induction; Induction$_{4d}$: expression after four days of induction; X: expression observed; -: no expression observed

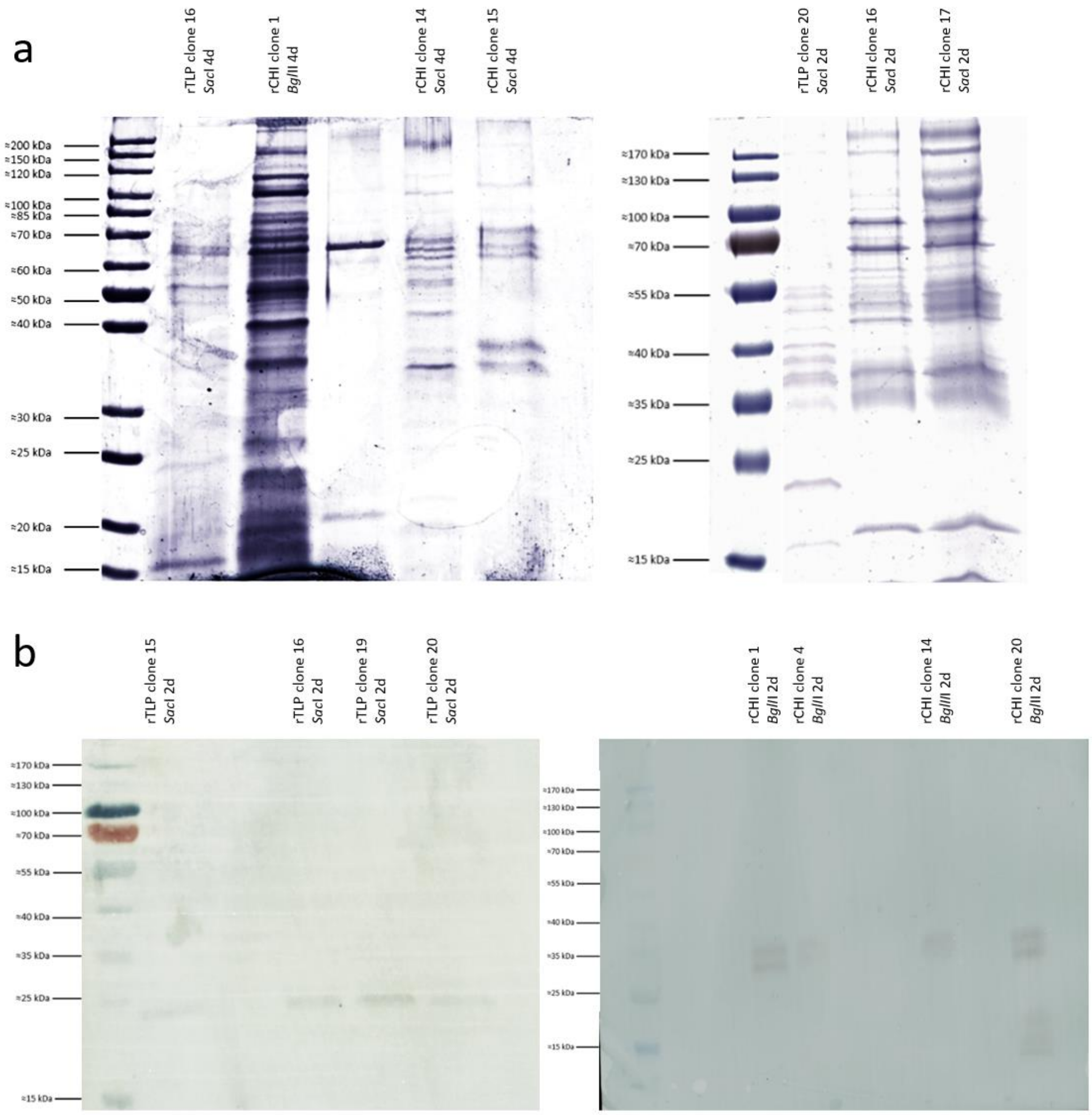

Figure S9: Protein expression of different clones (exemplary clones with visualized expression of 6x-his-tagged proteins) from the *Sac*I and *Bgl*II electroporation batches. a) SDS gel and b) WB blots showing the expression of rTLP (23-25 kDa) and rCHI (in the range of 27-37 kDa, double band).

2.4. Purification of the 6x-his-tagged rTLP and rCHI

2.4.1. Immobilized metal affinity chromatography (IMAC)

Cultures from the transformants able to express 6x-His-tagged proteins (confirmed by WB) were subjected to IMAC. A large peak (1) corresponding to the non-tagged proteins was first eluted (Figure S10 and S11), while the tagged proteins rTLP and rCHI could bind the column and where

later released by an isocratic flow of 100% of the elution buffer (containing imidazole), providing a peak (2) (Figure S10 and S11). Peak (2) was confirmed as rTLP (Figure S10) or rCHI (Figure S11) by SDS-PAGE gel and WB. A band at around 25 kDa could be recognized as rTLP (Figure S10) and the rCHI showed a double band between 25 kDa and 35 kDa (Figure S11).

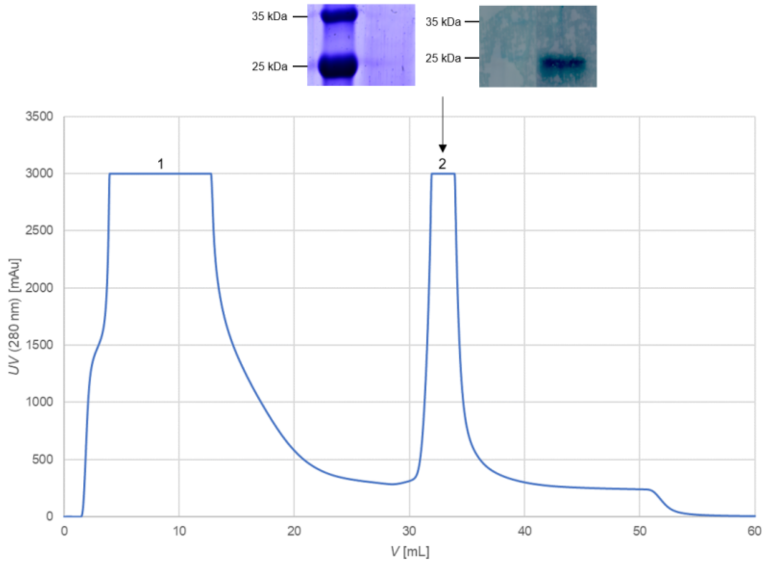

Figure S10: IMAC chromatogram of rTLP and visualization of the protein expression (peak 2) by SDS-PAGE gel and WB (protein markers are showed on the left side and the his-tagged rTLP on the right side).

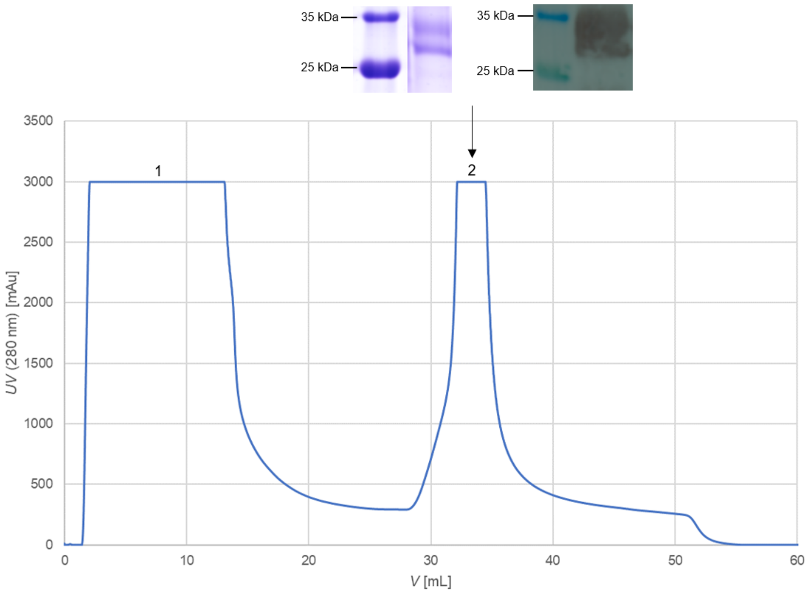

Figure S11: IMAC chromatogram of rCHI and visualization of the protein expression (peak 2) by SDS-PAGE gel and WB (protein markers are showed on the left side and the his-tagged rCHI on the right side).

2.4.2. Size Exclusion Chromatography (SEC)

By applying SEC, the samples were further purified from residual yeast proteins, which are still present after purification with IMAC. The SEC chromatogram in the Figure S12 shows the calibration of the column with small proteins (between 1.35 and 44 kDa) better separated than large proteins (between 158 and 670 kDa).

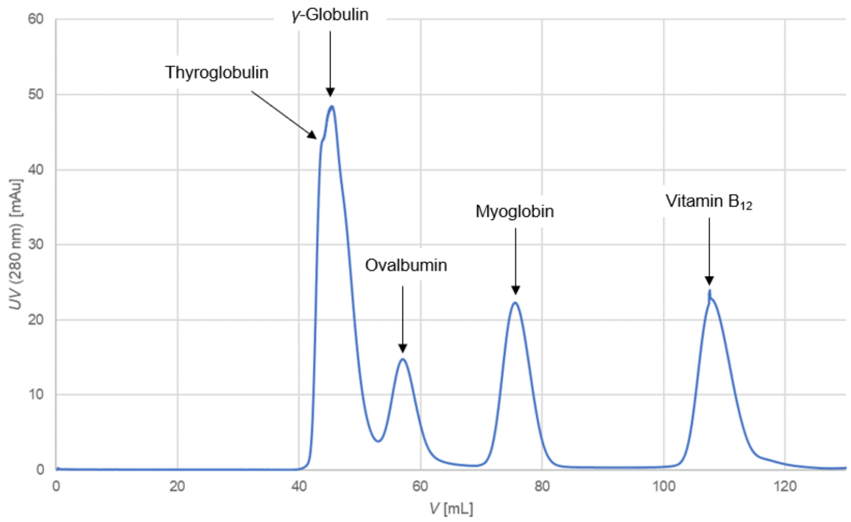

Figure S12: SEC chromatogram of the gel filtration standards separated by a HiLoad 16/600 Superdex 75 pg column.

Table S8: Molecular weight (MW) and elution volume (V_e) of the standard proteins.

Protein	MW [Da]	V_e [mL]
**Thyroglobulin**	670,000	43.11
**γ-Globulin**	158,000	45.28
**Ovalbumin**	44,000	57.12
**Myoglobin**	17,000	75.57
**Vitamin B12**	1,350	107.86

rTLP was collected between 70 to 75 mL of the eluted volume (MW range of 17-28 kDa). Fractions between 60 and 85 mL were also pooled and analyzed by SDS-PAGE and WB. Both proteins fractions showed a protein band in the range of 25 kDa (Figure S13).

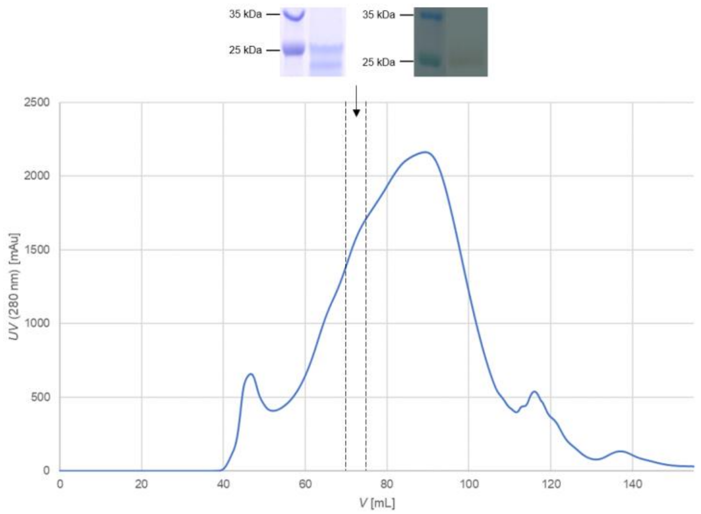

Figure S13: SEC chromatogram of rTLP and visualization of the protein expression (fraction eluted at 70-80 mL) by SDS-PAGE gel and WB.

rCHI showed a large peak between 60 and 80 mL (MW range of 15-40 kDa). Fractions between 60 and 90 mL were identified by SDS-PAGE and WB. Double bands at a height of 27-35 kDa could be recognized (Figure S14).

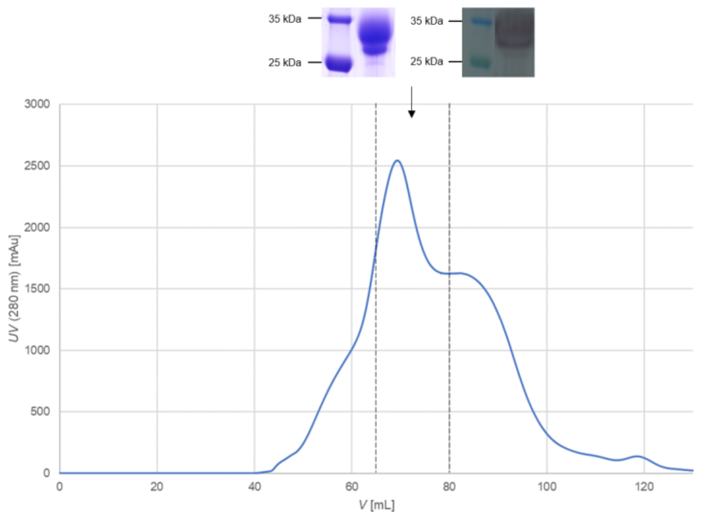

Figure S14: SEC chromatogram of rCHI and visualization of the protein expression (fraction eluted at 60-80 mL) by SDS-PAGE gel and WB.

2.5. MS-based bottom-up analysis

LC-MS proteomics analysis of the tryptic digested gel bands could unambiguously identify the expression of rTLP and rCHI.

2.5.1. rTLP

The analysis by the shotgun engine software found five peptides (5 unique peptides) corresponding to a TLP (PBD ID: 4JRU, UniProt ID: F6HUG9) (Figure S15 and Table S9).

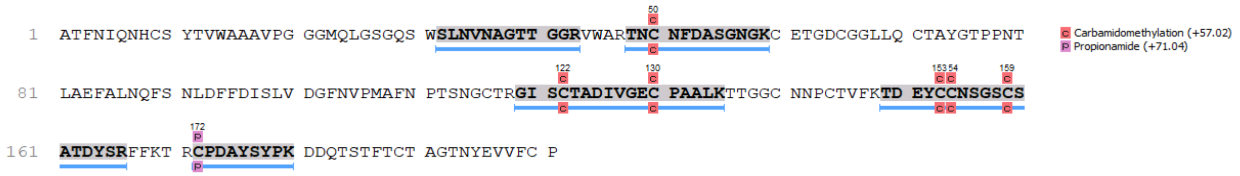

Figure S15: MS Identified peptides and chemical modification in the amino acid sequence of the rTLP. "c" and "p" are the carbamidomethylated or propionylated cysteine residues in the identified peptides.

Table S9: Identified protein and peptides from a MS-based analysis of the rTLP.

Accession	Coverage (%)	Unique peptides
F6HUG9	34	5
**Peptides**		
R.GISC(+57.02)TADIVGEC(+57.02)PAALK.T		
R.C(+71.04)PDAYSYPK.D		
R.TNC(+57.02)NFDASGNGK.C		
K.TDEYC(+57.02)C(+57.02)NSGSC(+57.02)SATDYSR.F		
W.SLNVNAGTTGGR.V		
		total 5 peptides

2.5.2. rCHI

For the chitinase class IV (UniProt ID: Q7XAU6) 25 peptides (8 unique peptides) were identified (Figure S16 and Table S10).

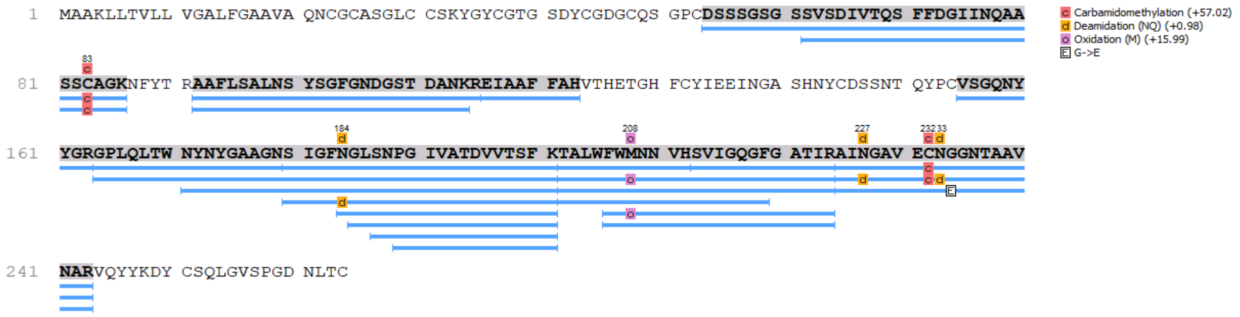

Figure S16: MS Identified peptides and chemical modification in the amino acid sequence of the rCHI. Additional chemical masses from carbamidomethylation "c", deamidation "d", oxidation "o" and gly -> glu substitution "G->E" are described as post translational modifications.

Table S10: Identified protein and peptides from a MS-based analysis of the rCHI.

Accession	Coverage (%)	Peptides
Q7XAU6	58	25
**Peptides**		
R.AAFLSALNSYSGFGNDGSTDANKR.E		
R.AAFLSALNSYSGFGNDGSTDANK.R		
R.AIN(+.98)GAVEC(+57.02)N(+.98)GGNTAAVNAR.V		
R.AINGAVEC(+57.02)NGGNTAAVNAR.V		
R.AINGAVECNE(sub G)GNTAAVNAR.V		
K.TALWFWM(+15.99)NNVHSVIGQGFGATIR.A		
K.TALWFWMNNVHSVIGQGFGATIR.A		
W.FWM(+15.99)NNVHSVIGQGFGATIR.A		
W.FWMNNVHSVIGQGFGATIR.A		
H.SVIGQGFGATIR.A		
K.TALWFWMNNVHSVIGQGF.G		
K.TALWFWMNNVH.S		
R.GPLQLTWNYNYGAAGNSIGFNGLSNPGIVATDVVTSFK.T		
W.NYNYGAAGNSIGFNGLSNPGIVATDVVTSFK.T		
N.SIGFN(+.98)GLSNPGIVATDVVTSFK.T		
N.SIGFNGLSNPGIVATDVVTSFK.T		
F.NGLSNPGIVATDVVTSFK.T		
N.GLSNPGIVATDVVTSFK.T		
L.SNPGIVATDVVTSFK.T		
N.PGIVATDVVTSFK.T		
C.DSSSGSGSSVSDIVTQSFFDGIINQAASSC(+57.02)AGK.N		
S.SVSDIVTQSFFDGIINQAASSC(+57.02)AGK.N		
R.GPLQLTWNYNYGAAGN.S		
C.VSGQNYYGR.G		
R.EIAAFFAH.V		
		**total 25 peptides**
		**8 unique peptides**

2.5.3. Glycosylation analysis (MS-analysis of rTLP and rCHI tryptic digests)

The Table S11 shows the identified peptides found in the glycosylation analysis for rTLP (in red) and rCHI (in blue). Only the peptide KDYCSQLGVSPGDNLTC from the rCHI was found to have the glycans $(Xyl)_1$-$(GlcNAc)_3$-$(Man)_4$ and $(Man)_3$ attached.

Table S11: Tryptic peptides from rTLP and rCHI identified in the MS-based glycosylation analysis.

rTLP	Peptides	Result
1	TVWAAASPGGGRR	No results found.
2	TNCNFDASGNGK	No results found.
3	TRCPDAYSYPK	No glycans with significant mass found.
4	LDSGQSWTITVNPGTTNAR	No glycans with significant mass found.
5	RLDSGQSWTITVNPGTTNAR	No glycans with significant mass found.
**rCHI**	**Peptides**	
1	SYSGFGNDGSTDANK	No glycans with significant mass found.
2	AINGAVECNGGNTAAVNAR	No glycans with significant mass found.
3	LSALNSYSGFGNDGSTDANK	No glycans with significant mass found.
4	NGLSNPGIVATDVVTSFK	No glycans with significant mass found.
5	KDYCSQLGVSPGDNLTC	- $(Xyl)_1$-$(GlcNAc)_3$-$(Man)_4$<br>- $(Man)_3$
6	SYSGFGNDGSTDANK	No glycans with significant mass found.

2.6. Chitinolytic activity

The results of the semi-quantitative chitinolytic activity estimated by the radius of the halos are shown in the Figure S17. The chitinolytic potential of the recombinant CHI (rCHI) was about 2 times higher than the activity of the CHI from the chitinase from *Streptomyces griseus* (cCHI), while the same samples pre-heated at served as a negative control (control). The denatured cCHI (95 °C for 10 min) did not show any zone of degradation, while the lyophilized protein content of a Silvaner Franken wine (SF), used as comparative control, which showed similar activity as the cCHI.

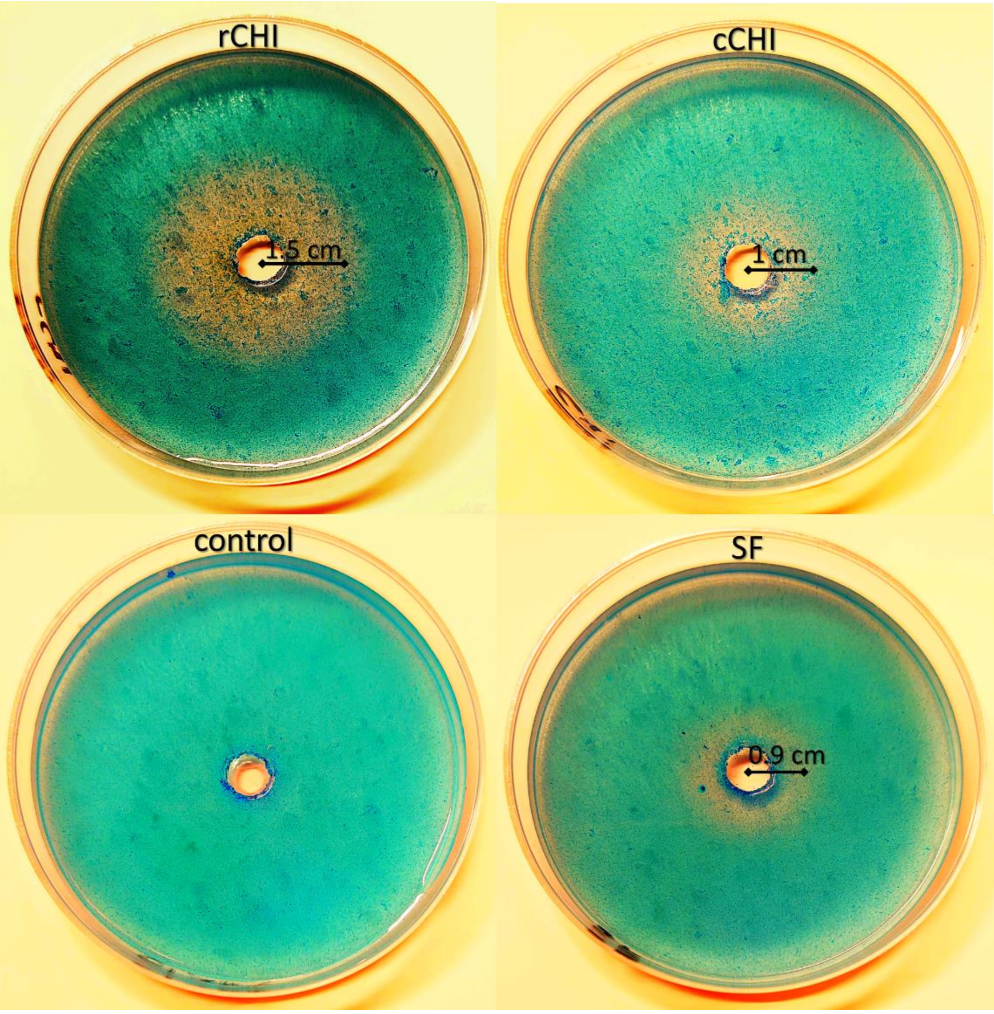

Figure S17: Chitinolytic activity of a recombinant CHI (rCHI), a commercial chitinase from *Streptomyces griseus* (cCHI). A pre-heated chitinase (denatured cCHI used as negative control) and chitinases present in proteins from a Silvaner Franken wine (SF). The radius of the halo of degradation is measured in cm and the chitin embedded in agar is stained (marine blue) after reaction with calcofluor white stain.

2.7. Analysis of the polyphenol extract

The chromatograms showing the polyphenols separations by HPLC are shown in the Figure S18. The identified polyphenolic compounds (Table S12) were caffeic acid (2.9 mg per gram of the grape juice dry extract); caftaric acid (3.9 mg/g); catechin (6.1 mg/g); coutaric acid (4.9 mg/g); epicatechin (10.7 mg/g); fertaric acid (1.1 mg/g); grape reaction product (GRP, 2-*S*-glutathionyl caftaric acid) (1.6 mg/g); *p*-coumaroyl–glucosyl–tartrate (*p*-CGT) (0.5 mg/g); procyanidin B1 (1.5 mg/g); procyanidin B2 (6.3 mg/g); procyanidin C1 (6.7 mg/g); protocatechuic acid (1.6 mg/g) and quercetin-3-*O*-glucoside (Que-3-glc) (0.7 mg/g).

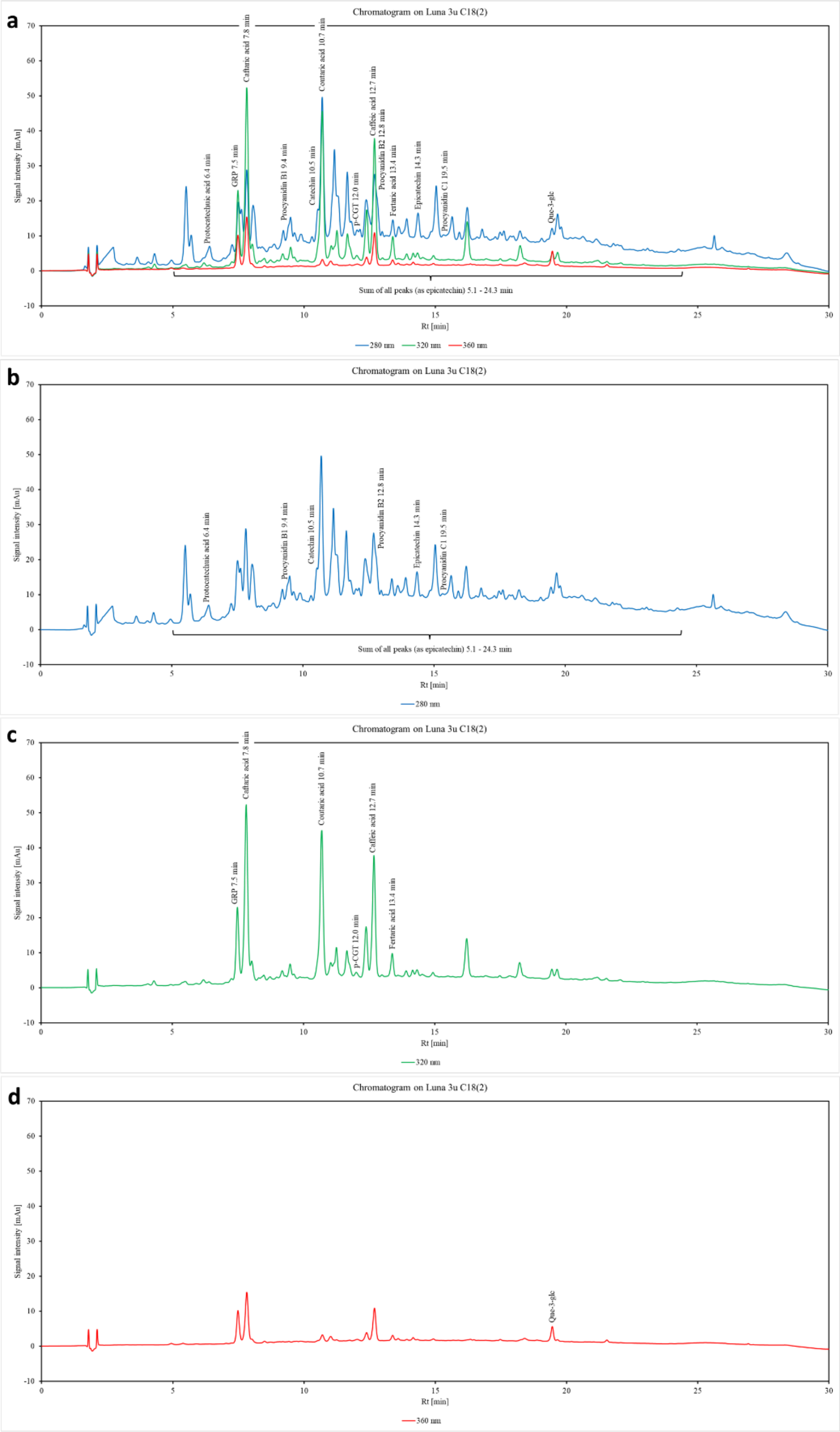

Figure S18: Separation of the polyphenolic compounds from white grape juice performed by UHPLC. The peaks were detected from 200 to 550 nm. The wavelengths b) 280, c) 320 and d) 360 nm were used for the evaluation and a) shows the overlay of all the chromatograms at different wavelengths.

Table S12: Wavelength (λ), retention time (Rt), area (A), concentration (c) and content of polyphenols contained in the extract.

Compound	λ [nm]	Rt [min]	A [mAu × min]	c [mg × L]	content [mg × g]
Protocatechuic acid	280	6.4	0.74	3.2	**1.6**
GRP	320	7.5	2.96	3.2	**1.6**
Caftaric acid	320	7.8	7.64	8.0	**3.9**
Procyanidin B1	280	9.4	0.69	3.1	**1.5**
Catechin	280	10.5	1.81	12.4	**6.1**
Coutaric acid	320	10.7	7.46	10.0	**4.9**
p-CGT	320	12.0	0.77	1.0	**0.5**
Caffeic acid	320	12.7	5.63	5.9	**2.9**
Procyanidin B2	280	12.8	1.46	12.9	**6.3**
Fertaric acid	320	13.4	1.66	2.2	**1.1**
Epicatechin	280	14.3	2.85	21.9	**10.7**
Procyanidin C1	280	15.3	1.07	13.8	**6.7**
Que-3-glc	360	19.5	0.57	1.5	**0.7**
Sum of identified peaks	–	–	35.31	99.1	**48.4**
Sum of all peaks (as epicatechin)	280	5.1–24.3	124.78	950.50	**464.3**

The monosaccharide content in the polyphenol extract was determined and their total mass corresponded to 17.97%. Table S13 shows the retention times, the mass fraction and the standard deviation of the monosaccharides in the measured polyphenol extract. Figures S19 and S20 show the chromatograms of the neutral and acidic monosaccharides present in the polyphenol extract, respectively.

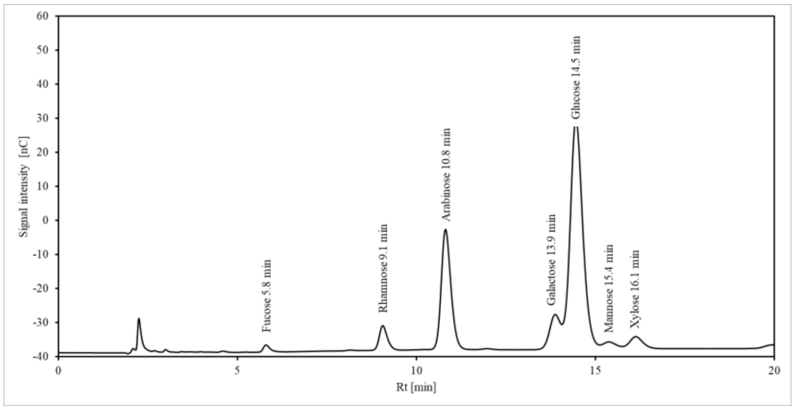

Figure S19: Chromatograms of the neutral monosaccharides present in the polyphenol extract.

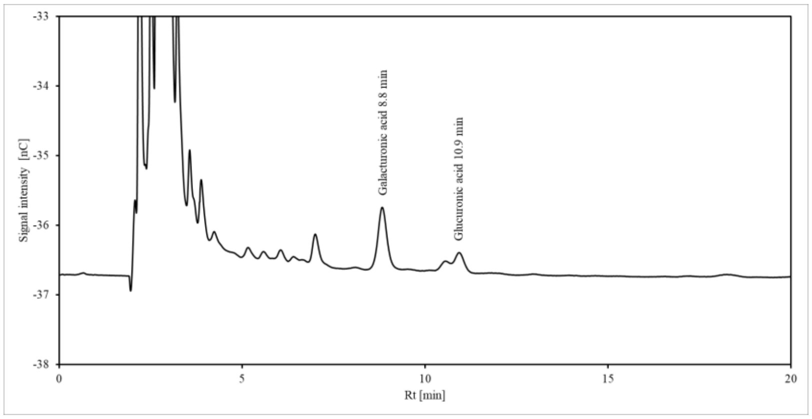

Figure S20: Chromatograms of the acidic monosaccharides present in the polyphenol extract.

Table S13: Retention time (Rt), mass fraction and standard deviation (σ) of the monosaccharides in the extract (values marked in gray were below the concentration of the smallest standard).

Monosaccharide	Rt [min]	mass fraction [%]	σ [%]
Fucose	5.8	**0.21**	0.01
Rhamnose	9.1	**1.17**	0.05
Arabinose	10.8	**4.45**	0.25
Galactose	13.9	**1.20**	0.05
Glucose	14.5	**9.58**	0.29
Mannose	15.4	**0.41**	0.02
Xylose	16.1	**0.54**	0.05
Galacturonic acid	8.8	**0.34**	0.02
Glucuronic acid	10.9	**0.06**	0.00
**total**	–	**17.97**	0.72

2.8. Comparison of the haze potential of the rTLP and rCHI and the haze potential of a wine protein solution (Silvaner Franken wine)

To compare the experimental conditions of the rTLP and rCHI with proteins obtained from real wines, colloids of a Silvaner Franken wine (as published in https://doi.org/10.1016/j.foodchem.2021.130437) was used for comparative analysis. The wine colloids were able to form haze similarly to the recombinant proteins. Protein aggregation was strikingly induced by sulfite ions (at 1 mg/mL) and by the polyphenol extracts (at both concentrations of 0.25 mg/mL and 0.5 mg/mL). Similar results obtained with the experiments with rTLP and rCHI show that recombinant proteins could substitute the native TLP and CHI from *V. vinifera* in experimental conditions. Haze levels and the pellets formed and absorbances of heat tests using proteins from a Silvaner Franken wine can be visualized in the Figure S21.

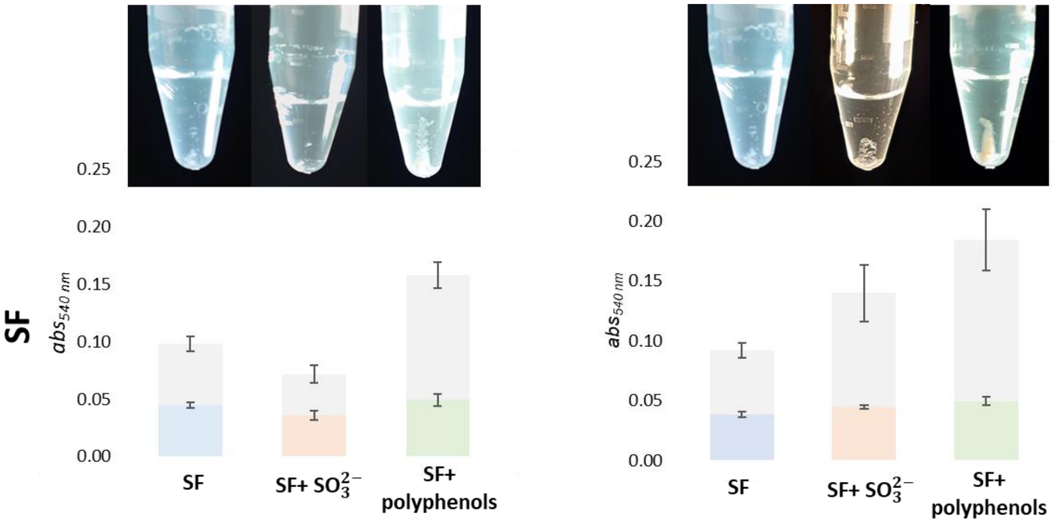

	SF protein content 0.1 mg/mL		SO_3^{2-} 0.5 mg/mL		+polyphenols 0.25 mg/mL	
	Δabs	σ	Δabs	σ	Δabs	σ
SF	0.054	0.001	0.036	0.004	0.109	0.011

	SF protein content 0.1 mg/mL		SO_3^{2-} 1 mg/mL		+polyphenols 0.5 mg/mL	
	Δabs	σ	Δabs	σ	Δabs	σ
SF	0.054	0.001	0.095	0.001	0.135	0.003

Figure S21: Influence of SO_3^{2-} and polyphenols in the protein aggregation and haze levels of a protein solution from a Silvaner Franken wine. Aggregation levels are showed in terms of absorbance (in bars) and the visible residual pellet formed (at the bottom of tubes) and the experimental variants are divided into SF protein content, added by sulfite ions (SO_3^{2-}) and added by polyphenols. The gray segment of each bar represents the absorbance after the heat test (haze formation) and the colored part the final absorbance after centrifugation. The columns on the left and right display respectively the experiments with lower and higher concentration of matrix compounds (SO_3^{2-} and polyphenols). The tables show the values of formed haze (at 540 nm) in the different experimental conditions and the highlighted table (outlined by a blue line) shows the values at higher concentrations of sulfite ions and polyphenols.

2.9. Bentonite treatment of the rTLP and rCHI

All the concentrations of bentonite tested (0.25, 0.5 and 1 g/L) were able to precipitate both rTLP and rCHI and form a residual pellet on the bottom of the vials after centrifugation. A heat test performed for all the samples showed no haze formation after the bentonite fining (Figure S22b). Figure S22a show the samples directly after mixing with bentonite and Figure S22b after centrifugation and heating.

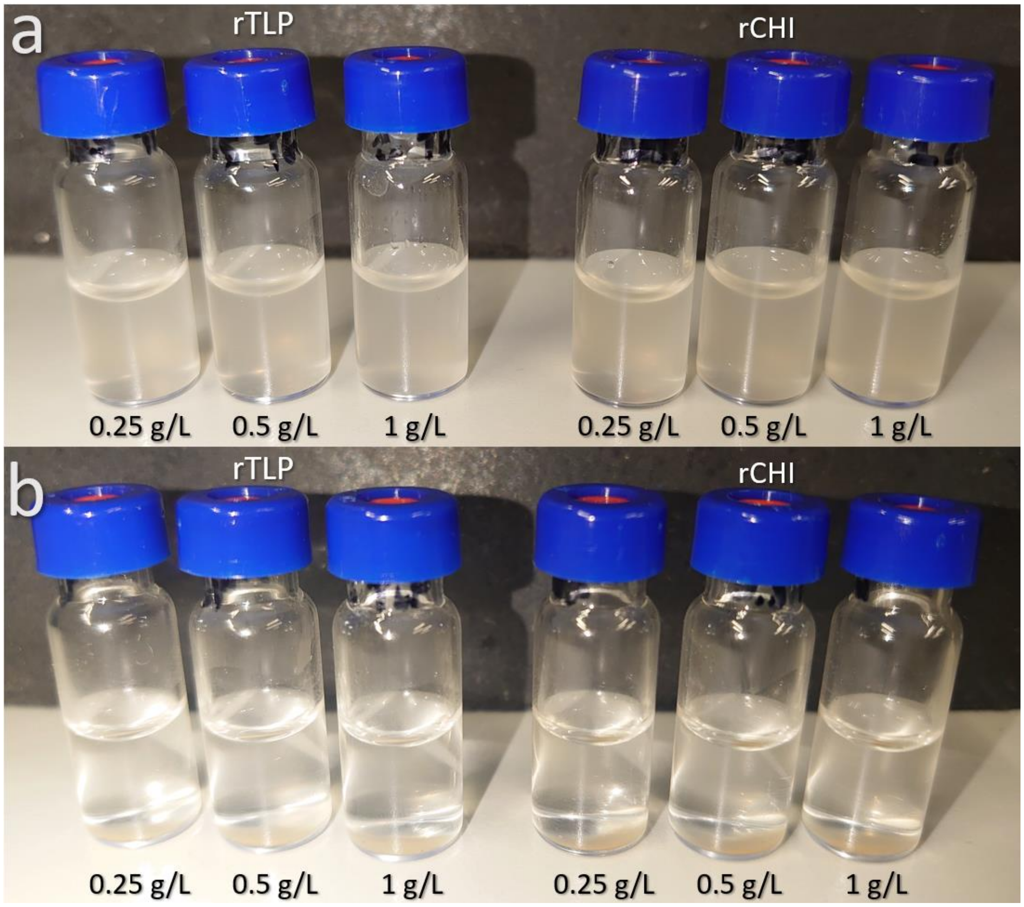

Figure S22: Heat test performed after a bento test (bentonite concentrated at 0.25, 0.5 and 1 g/L) using the rTLP and rCHI in model solutions (citrate buffer, at pH 4). a) rTLP and rCHI mixed with bentonite power in three different concentrations. B) rTLP and rCHI after 2 h of rest, centrifugation, and a subsequent heat test.

3. References

1. Bradford, M.M. A Rapid and Sensitive Method for the Quantitation of Microgram Quantities of Protein Utilizing the Principle of Protein-Dye Binding. *Anal Biochem.* **1976**, *72*, 248–254, doi:10.1016/0003-2697(76)90527-3.

2. U. K. Laemmli Cleavage of Structural Proteins during the Assembly of the Head of Bacteriophage T4. *Nature* **1970**, *227*, 680–685.

3. Trudel, J.; Asselin, A. Detection of Chitin Deacetylase Activity after Polyacrylamide Gel Electrophoresis. *Anal Biochem.* **1990**, *189*, 249–253, doi:10.1016/0003-2697(90)90116-Q.

4. Zou, X.; Nonogaki, H.; Welbaum, G.E. A Gel Diffusion Assay for Visualization and Quantification of Chitinase Activity. *Mol. Biotechnol.* **2002**, *22*, 019–024, doi:10.1385/MB:22:1:019.

5. Breuil, C.; Saddler, J.N. Comparison of the 3,5-Dinitrosalicylic Acid and Nelson-Somogyi Methods of Assaying for Reducing Sugars and Determining Cellulase Activity. *Enzyme Microb. Technol.* **1985**, *7*, 327–332, doi:10.1016/0141-0229(85)90111-5.

6. Brandt, S.C.; Ellinger, B.; van Nguyen, T.; Thi, Q.D.; van Nguyen, G.; Baschien, C.; Yurkov, A.; Hahnke, R.L.; Schäfer, W.; Gand, M. A Unique Fungal Strain Collection from Vietnam Characterized for High Performance Degraders of Bioecological Important Biopolymers and Lipids. *PLoS One* **2018**, *13*, e0202695, doi:10.1371/journal.pone.0202695.

7. Pocock, K.F.; Salazar, F.N.; Waters, E.J. The Effect of Bentonite Fining at Different Stages of White Winemaking on Protein Stability. *Aust. J. Grape Wine Res.* **2011**, *17*, 280–284, doi:10.1111/j.1755-0238.2011.00123.x.

8. Pocock, K.F.; Waters, E.J. Protein Haze in Bottled White Wines: How Well Do Stability Tests and Bentonite Fining Trials Predict Haze Formation during Storage and Transport? *Aust. J. Grape Wine Res.* **2006**, *12*, 212–220, doi:10.1111/j.1755-0238.2006.tb00061.x.
